# GADD45a and GADD45b Genes in Rheumatoid Arthritis and Systemic Lupus Erythematosus Patients

**DOI:** 10.3390/jcm8060801

**Published:** 2019-06-05

**Authors:** Ruei-Nian Li, Yuan-Zhao Lin, Ya-Chun Pan, Chia-Hui Lin, Chia-Chun Tseng, Wan-Yu Sung, Cheng-Chin Wu, Tsan-Teng Ou, Wen-Chan Tsai, Jeng-Hsien Yen

**Affiliations:** 1 Department of Biomedical Science and Environmental Biology, College of Life Science, Kaohsiung Medical University, Kaohsiung 807, Taiwan; runili@kmu.edu.tw; 2 Graduate Institute of Clinical Medicine, College of Medicine, Kaohsiung Medical University, Kaohsiung 807, Taiwan; sonyauron@gmail.com (Y.-Z.L.); pyc79082@yahoo.com.tw (Y.-C.P.); judyann082000@gmail.com (C.-H.L.); 3 Division of Rheumatology, Department of Internal Medicine, Kaohsiung Medical University Hospital, Kaohsiung 807, Taiwan; 990331kmuh@gmail.com (C.-C.T.); hemidark@gmail.com (W.-Y.S.); wucc@kmu.edu.tw (C.-C.W.); tsanteng@kmu.edu.tw (T.-T.O.); d740094@kmu.edu.tw (W.-C.T.); 4 Institute of Biomedical Sciences, National Sun Yat-Sen University, Kaohsiung 807, Taiwan; 5 Department of Biological Science and Technology, National Chiao Tung University, Hsinchu 300, Taiwan

**Keywords:** GADD45a and GADD45b genes, rheumatoid arthritis, systemic lupus erythematosus

## Abstract

Background: GADD45 genes are stress sensors in response to cellular stress response, activated signal pathways leading to the stimulation of inflammatory cytokines. This study is to examine the associations of GADD45a and GADD45b genes with rheumatoid arthritis (RA) and systemic lupus erythematosus (SLE) patients. Methods: 230 patients of RA, 140 patients of SLE, and 191 healthy controls were enrolled. Genomic DNA was extracted from peripheral blood mononuclear cells and gene polymorphisms were genotyped by TaqMan assay. RNA expression was quantitated with real-time polymerase chain reaction. Results: The RNA expression of the GADD45b gene was significantly lower in RA patients than the control cases (*p* = 0.03). The odds ratio of GADD45a genotype -589 CC (rs581000) was significantly low (OR = 0.36, 95% CI, 0.15–0.87) in DR4-negative RA patients. The odds ratio of GADD45b genotype -712CT (rs3795024) in DR4-negative RA patients was 0.41 (95% CI, 0.18–0.95). In clinical manifestation, the odds ratio of GADD45b -712CT genotype with anti-RNP antibody was 4.14 (95% CI, 1.10–15.63) in SLE patients. GADD45a genotype -589GG+GC was associated with rheumatoid factor (RF) in SLE patients. Conclusions: Genotypes GADD45a -589CC and GADD45b -712CT were shown to be less susceptible to RA and related to the disease state in SLE patients.

## 1. Introduction

Growth arrest and DNA damage-inducible 45 (GADD45) family members include GADD45a, GADD45b and GADD45g genes. GADD45 members are highly acidic, small proteins and are stress sensors in response to cellular stress response [1,2,3]. GADD45 family proteins are mainly produced by hematopoietic cells [4]. Previous reports showed that GADD45 family proteins inhibited the proliferation of cells by directly interacting with cell cycle proteins [5] and promoted apoptosis by acting on JNK/p38 MAP kinases [6]. GADD45a and GADD45b proteins showed to play modulation roles in innate immunity [7,8]. Deficiency of GADD45 family genes caused malfunctions of innate immune response, such as oxidative burst, reactive oxygen species production and phagocytosis [3,9]. GADD45a was involved in MAPK kinase cascade through MKK3/6 to activate p38 pathway in granulocytes [10,11]. GADD45a gene knockout mice developed human like systemic lupus erythematosus (SLE) [12]. GADD45b was induced in CD4^+^ T cells by inflammatory cytokines, such as IL-12 and IL-18 [7,13]. GADD45b could suppress B cell apoptosis in response to Fas stimulation through the activation of NF-κB [14]. In macrophage, GADD45b exerted on MAPK kinase cascade through MKK4/7 to activate JNK pathway [11]. Previous reports also demonstrated that GADD45a-deficient and GADD45b-deficient mice were defective in the recruitment of polymorphic mononuclear cells in the mouse sepsis model [11].

Cytokines are well known for their roles in controlling immune responses, inflammatory mediators, cell proliferation as well as in the development of aberrant inflammation [15]. Signaling pathways of cytokines are involved in the pathogenesis of autoimmune diseases [16]. GADD45 genes are stress sensors in response to the cellular stress response, and activate pathways leading to the stimulation of a variety of cytokines and inflammatory substances [7,13]. Pro-inflammatory cytokines, like IL-6, IL-8, IL-18 and TNF are involved in the inflammatory process of autoimmune diseases, such as rheumatoid arthritis (RA) and SLE [16,17,18]. RA and SLE are complex, chronic systemic inflammation diseases that may involve extra-articular organs in addition to joints and multiple organs, respectively. However, the etiological agent of these diseases is still unclear [19]. It is important to elucidate the roles of GADD45 family genes in the immunopathogenesis of autoimmune diseases. Therefore, in this study, we have carried out experiments to examine the gene polymorphisms and gene expressions of GADD45a and GADD45b genes in RA and SLE patients.

## 2. Materials and Methods

### 2.1. Clinical Subjects

Two hundred and thirty (179 female, 51 male) patients of RA, 140 patients of SLE (129 female, 11 male) and 191 healthy controls (113 female, 78 male) were enrolled in their recruitment from the Kaohsiung Medical University Hospital. Age in the control group is matched with RA and SLE patients. The diagnosis of RA or SLE was according to the criteria set of the American College of Rheumatology and European League Against Rheumatism (ACR/EULAR) for the classification of autoimmune diseases [20]. This study was approved by the Institutional Review Board of Kaohsiung Medical University Hospital (KMUH).

### 2.2. Genomic DNA and Total RNA Extraction

Peripheral blood mononuclear cells (PBMC) were collected by Ficoll-paque (GE Healthcare) purification and genomic DNA was extracted from PBMC of collected patients from KMUH. Total RNA was extracted by the method of QIAmp RNA Blood Mini Kit (Qiagen, Hilden, Germany). RNA was reversely transcribed to cDNA with random primers and high capacity cDNA Archive kit (Applied Biosystems, Life Technologies, Waltham, MA, USA).

### 2.3. SNP Genotyping and Data Analysis

Three different polymorphic sites on the promoter region of the GADD45a and GADD45b genes were genotyped. The GADD45a and GADD45b SNPs genotyping were performed by TaqMan SNP Genotyping Assays (Applied Biosystems, Life Technologies) by the real-time polymerase chain (PCR) reaction with ABI 7500 Real-Time PCR systems. The odds ratio (OR) and its 95% confidence interval (CI) were calculated to evaluate the risk of disease. The statistical analysis was conducted by the IBM SPSS Statistics version 19 or Fisher exact test.

### 2.4. GADD45a and GADD45b RNA Expression

A total of 91 RA patients, 75 SLE patients and 80 control individuals were assayed for the GADD45a and GADD45b expression. The RNA expression was performed by the TaqMan Gene Expression Assay (Applied Biosystems, cat. no. 4369016) and real-time PCR reaction mix (Applied Biosystems, cat. no. 4369016). The reverse transcription was at 50 °C for 2 min, PCR condition was at 95 °C for 10 min, 95 °C for 10 s, 60 °C for 1 min (repeat 40 cycles) on ABI 7500 Real-Time PCR systems. RNA polymerase II is the input control [21].

## 3. Results

### 3.1. RNA Expression of GADD45b Was Lower in RA Patients

We performed the TaqMan real-time polymerase chain reaction to evaluate the RNA expression of GADD45a and GADD45b genes in RA and SLE patients. GADD45b RNA expression was 1.21 ± 0.71 in healthy cases and was 0.99 ± 0.56 in RA patients. RNA expression of GADD45b gene was significantly lower in RA patients than the control cases (Table 1). In contrast, GADD45a gene expression in RA patients, and GADD45a, GADD45b genes expression in SLE patients had no statistical difference with control cases (Table 1).

### 3.2. GADD45a -589CC and GADD45b -712CT Genotypes are Less Susceptible to Rheumatoid Arthritis in DR4-Negative Individuals

Previous study has demonstrated that the GADD45 members were associated with the susceptibility to autoimmune diseases [12]. Therefore, in this study, we have carried out experiments to determine polymorphic sites on promoter regions of GADD45a and GADD45b genes in RA and SLE patients. Genotype -589G/C (rs581000) of GADD45a promoter sequence was not related to the susceptibility of patients with RA or SLE (Table 2). We also determined polymorphic sites on promoter region -1539A/G (rs115517134) and 343G/A (rs11544978) in exon 1 of GADD45a gene in patients and were not found in relation to RA or SLE. Genotypes of GADD45b promoter sequence -712C/T (rs3795024), -438C/A (rs3729535) (Table 3) and 459C/G (rs11541535) were also unrelated to the susceptibility of patients with RA or SLE. However, the odds ratio of GADD45a -589 C/C (rs581000) genotype was 0.36 (95% CI, 0.15–0.87) in DR4-negative RA patients (Table 4). The odds ratio of GADD45b -712C/T (rs3795024) genotype was 0.41 (95% CI, 0.18–0.95) in DR4-negative RA patients (Table 4).

### 3.3. GADD45b -712CT and GADD45a -589GG+CC Genotypes are Associated with Clinical Features of SLE Patients

In clinical manifestation, the odds ratio of GADD45b -712CT genotype with anti-RNP antibodies was 4.14 (95% CI, 1.10–15.63) in SLE patients, 11 out of 14 patients with anti-ribonucleoprotein (RNP) antibody were -712CT (Table 5). GADD45a genotype -589GG+GC was associated with rheumatoid factor (RF) in SLE patients. None of the SLE patient with genotype -589CC was RF-positive (Table 6).

## 4. Discussion

Promoter polymorphism has been demonstrated to be associated with different inflammatory diseases, such as RA, SLE, Sjögren’s syndrome [21,22,23]. We have investigated different polymorphic sites on the promoter and exon 1 of GADD45a and GAdd45b genes in RA and SLE patients. The results indicated that the promoter and exon 1 genotypes of GADD45a and GADD45b genes were found in no relation to the susceptibility with RA or SLE patients. Of the tested GADD45a and GADD45b genes promoter and exon 1 genotypes, there was no correlation with RA or SLE patients. However, we noticed that the genotypes GADD45a -589CC (rs581000) and GADD45b -712CT (rs3795024) were associated with the susceptibility of RA in DR4-negative individuals. The odds ratio of GADD45a -589CC (rs581000) genotype was 0.36 in DR4-negative RA patients. GADD45a -589CC genotype is associated with protection against rheumatoid arthritis in DR4-negative individuals. The results suggested that the G allele was the risk factor, whereas the C allele could be less susceptible to rheumatoid arthritis. GADD45a promoter region -589G/G or C/G (rs581000) genotypes could prevent rheumatoid arthritis. The odds ratio of GADD45b -712CT genotype was 0.41 in DR4-negative RA patients. The results indicated the protective effect of T allele of -712CT in RA patients’ disease condition. RNA expression of GADD45b gene was significantly lower in RA patients, but not GADD45a. The GADD45a and GADD45b genes expression of SLE patients had no statistical difference with control cases. We have also characterized other lupus symptoms such as, malar rash, optic neuritis, lupus headache, photosensitivity, serositis, anti-dsDNA, anti-Sm antibodies, leukopenia and thrombocytopenia. We assayed the correlations of these lupus symptoms with the GADD45a and GADD45b genotypes, but the results were not significant. We checked the clinical features and the findings were not significant as expected maybe partly due to the small number of sample size. In the future studies, we will include a large number of samples, the study can be improved and the findings of disease phenotype may be more significant. In contrast, the GADD45a and GADD45b genotypes had significant difference in odds ratios of anti-RNP antibodies and RF, respectively, in SLE patients. In the majority of SLE patients, the GADD45b -712CT genotypes associated with anti-RNP antibodies in SLE patients. The GADD45a genotype -589GG+GC was also associated with RF in SLE patients. The T allele of -712CT or C allele of -589GC contributing alleviative effect is still in question, it is required for further evidences to confirm the disease severity.

Cytokines are involved in the pathogenesis of autoimmune diseases [16,24]. GADD45a or GADD45b acts on the MAPK kinase cascade through either MKK4/7 to activate p38 or JNK pathways, respectively [11]. Subsequently, GADD45a and GADD45b activate and stimulate the production of various cytokines and inflammatory factors [25,26,27]. Notably, we noticed that the RNA expression of GADD45b was lower in RA patients than the healthy cases. GADD45b was secreted in synovial fluid of RA patients as previously reported [28]. Further experiments are needed to elucidate the level of GADD45b as expressed in the serum of RA patients.

RNA expression of GADD45b was associated with the cytokine production and T helper cell differentiation [29,30], the GADD45a and GADD45b genes also regulate gene methylation [31,32]. In conclusion, we have shown that GADD45b was expressed in lower amount of RNA in RA patients. We have also identified that -589 CC (rs581000) genotype of GADD45a was less susceptible to RA in DR4-negative individuals. The disease severity of RA can be partially explained by the GADD45a polymorphisms and GADD45b expression. GADD45 gene members are playing important regulatory roles in the pathogenesis of autoimmune diseases. It is important and necessary to determine the regulatory roles of the GADD45 gene family in the pathogenesis of autoimmune diseases in further studies.

## Figures and Tables

**Table 1:** The RNA expression of GADD45a and GADD45b in rheumatoid arthritis (RA) and systemic lupus erythematosus (SLE) patients.

GADD45a Expression	RA (*n* = 91)	Control (*n* = 80)	*p*-Value
Expression level (2-ΔCT)	0.075 ± 0.04	0.077 ± 0.045	NS
Expression level (2-ΔCT)	**SLE (*n* = 75)**	**Control (*n* = 80)**	***p*-Value**
	0.081 ± 0.04	0.077 ± 0.045	NS
**GADD45b Expression**	**RA (*n* = 91)**	**Control (*n* = 80)**	***p*-Value**
Expression level (2-ΔCT)	0.99 ± 0.56 (*n* = 91)	1.21±0.71 (*n* = 80)	0.03
Expression level (2-ΔCT)	**SLE (*n* = 75)**	**Control *n* = 80)**	***p*-Value**
	1.32 ± 0.72 (*n* = 75)	1.21 ± 0.71 (*n* = 80)	NS

**Table 2:** Genotypes frequency of -589G/C GADD45a gene in RA and SLE patients.

**GADD45a Genotype**	**-589 G/C (rs581000)**	**RA (*n* = 230)**	**Control (*n* = 191)**	**OR**
		***n*(%)**	***n* (%)**	**95% CI**
	GG	79 (34.35%)	70 (36.65%)	1
	CG	119 (51.74%)	89 (46.60%)	1.18 (0.78~1.81)
	CC	32 (13.91%)	32 (16.75%)	0.89 (0.49~1.59)
	**-589 G/C (rs581000)**	**SLE (*n* = 140)**	**Control (*n* = 191)**	**OR**
		***n* (%)**	***n* (%)**	**95% CI**
	GG	65 (46.43%)	70 (36.65%)	1
	CG	56 (40.00%)	89 (46.60%)	0.68 (0.42~1.09)
	CC	19 (13.57%)	32 (16.75%)	0.64 (0.33~1.24)

OR: odds ratio. CI: confidence interval.

**Table 3:** Genotypes frequency of -712C/T, -438C/A GADD45b gene in RA and SLE patients.

**GADD45b Genotype**	**-712 C/T (rs3795024)**	**RA (*n* = 230)**	**Control (*n* = 191)**	**OR**
		***n* (%)**	***n* (%)**	**95% CI**
	CC	206 (89.57%)	162 (84.82%)	1
	CT	24 (10.43%)	29 (15.18%)	0.65 (0.36~1.16)
	**-712 C/T (rs3795024)**	**SLE (*n* = 140)**	**Control (*n* = 191)**	**OR**
		***n* (%)**	***n* (%)**	**95% CI**
	CC	126 (90.00%)	162 (84.82%)	1
	CT	14 (10.00%)	29 (15.18%)	0.62 (0.31~1.22)
**GADD45b Genotype**	**-438 C/A (rs3729535)**	**RA (*n* = 230)**	**Control (*n* = 191)**	**OR**
		***n* (%)**	***n* (%)**	**95% CI**
	CC	205 (89.13%)	164 (85.86%)	1
	CA	25 (10.87%)	27 (14.14%)	0.74 (0.41~1.32)
	**-438 C/A (rs3729535)**	**SLE (*n* = 140)**	**Control (*n* = 191)**	**OR**
		***n* (%)**	***n* (%)**	**95% CI**
	CC	127 (90.71%)	164 (85.86%)	1
	CA	13 (9.29%)	27 (14.14%)	0.62 (0.31~1.25)

OR: odds ratio. CI: confidence interval.

**Table 4:** Genotype frequency of -589G/C GADD45a, -712C/T GADD45b in DR4-positive and DR4-negative RA patients.

	DR4 Positive					DR4 Negative			
	GADD45a Genotype	RA (*n* = 103)	Control (*n* = 20)	OR	*p*-Value	RA (*n* = 122)	Control (*n* = 89)	OR	*p*-Value
		*n* (%)	*n* (%)	95% CI		*n* (%)	*n* (%)	95% CI	
**-589 G/C (rs581000)**	GG + GC	82 (79.61%)	18 (90.00%)	1	NS	113 (92.62%)	73 (82.02%)	1	0.029
	CC	21 (20.39%)	2 (10.00%)	2.3 (0.5~10.72)		9 (7.38%)	16 (17.98%)	0.36 (0.15~0.87)	
	**DR4 Positive**					**DR4 Negative**			
	**GADD45b Genotype**	**RA (*n* = 103)**	**Control (*n* = 20)**	**OR**	***p*-Value**	**RA (*n* = 122)**	**Control (*n* = 89)**	**OR**	***p*-Value**
		***n* (%)**	***n* (%)**	**95% CI**		***n*(%)**	***n* (%)**	**95% CI**	
**-712 C/T (rs3795024)**	CC	90 (87.37%)	19 (95%)	1	NS	112 (91.8%)	73 (82.02%)	1	0.033
	CT	13 (12.63%)	1 (5%)	2.74 (0.34~1.21)		10 (8.2%)	16 (17.98%)	0.41 (0.18~0.95)	

OR: odds ratio. CI: confidence interval.

**Table 5:** Genotypes frequency of -712C/T, GADD45b gene in anti-RNP Ab positive and anti-RNP Ab negative SLE patients.

	GADD45b Genotype	Anti-RNP Ab Positive (*n* = 65)	Anti-RNP Ab Negative (*n* = 64)	OR	*p-*Value
		*n* (%)	*n* (%)	95% CI	
**-712C/T (rs3795024)**	CC	54 (83.08%)	61 (95.31%)	1	0.044
	CT	11 (16.92%)	3 (4.69%)	4.14 (1.10~15.63)	

RNP: ribonucleoprotein. Ab: antibody. CI: confidence interval.

**Table 6:** Genotypes frequency of -589G/C GADD45a gene in RF-positive and RF-negative SLE patients.

	GADD45a Genotype	RF Positive (*n* = 18)	RF Negative (*n* = 44)	OR	*p-*Value
		*n* (%)	*n* (%)	95% CI	
**-589 G/C (rs581000)**	GG + GC	18 (100%)	32 (72.73%)	1	0.01
	CC	0 (0%)	12 (27.27%)	0.07 (0.004~1.27)	

RF: rheumatoid factor. CI: confidence interval.

## References

[1] Takekawa M., Saito H. (1998). A Family of Stress-Inducible Gadd45-Like Proteins Mediate Activation of the Stress-Responsive Mtk1/Mekk4 Mapkkk. Cell.

[2] Amanullah A., Azam N., Balliet A., Hollander C., Hoffman B., Fornace A., Liebermann D. (2003). Cell Signalling: Cell Survival and a Gadd45-Factor Deficiency. Nature.

[3] Hoffman B., Liebermann D.A. (2009). Gadd45 Modulation of Intrinsic and Extrinsic Stress Responses in Myeloid Cells. J. Cell. Physiol..

[4] Gupta S.K., Gupta M., Hoffman B., Liebermann D.A. (2006). Hematopoietic Cells from Gadd45a-Deficient and Gadd45b-Deficient Mice Exhibit Impaired Stress Responses to Acute Stimulation with Cytokines, Myeloablation and Inflammation. Oncogene.

[5] Zhan Q., Antinore M.J., Wangm X.W., Carrier F., Smith M.L., Harris C.C., Fornace A.J. (1999). Association with Cdc2 and Inhibition of Cdc2/Cyclin B1 Kinase Activity by the P53-Regulated Protein Gadd45. Oncogene.

[6] Hildesheim J., Bulavin D.V., Anver M.R., Alvord W.G., Hollander M.C., Vardanian L., Fornace A.J. (2002). Gadd45a Protects against Uv Irradiation-Induced Skin Tumors, and Promotes Apoptosis and Stress Signaling Via Mapk and P53. Cancer Res..

[7] Lu B., Ferrandino A.F., Flavell R.A. (2004). Gadd45beta Is Important for Perpetuating Cognate and Inflammatory Signals in T Cells. Nat. Immunol..

[8] Jirmanova L., Jankovic D., Fornace A.J., Ashwell J.D. (2007). Gadd45alpha Regulates P38-Dependent Dendritic Cell Cytokine Production and Th1 Differentiation. J. Immunol..

[9] Lu B. (2006). The Molecular Mechanisms That Control Function and Death of Effector Cd4+ T Cells. Immunol. Res..

[10] Salvador J.M., Mittelstadt P.R., Belova G.I., Fornace A.J., Ashwell J.D. (2005). The Autoimmune Suppressor Gadd45alpha Inhibits the T Cell Alternative P38 Activation Pathway. Nat. Immunol..

[11] Salerno D.M., Tront J.S., Hoffman B., Liebermann D.A. (2012). Gadd45a and Gadd45b Modulate Innate Immune Functions of Granulocytes and Macrophages by Differential Regulation of P38 and Jnk Signaling. J. Cell. Physiol..

[12] Salvador J.M., Hollander M.C., Nguyen A.T., Kopp J.B., Barisoni L., Moore J.K., Ashwell J.D., Fornace A.J. (2002). Mice Lacking the P53-Effector Gene Gadd45a Develop a Lupus-Like Syndrome. Immunity.

[13] Yang J., Zhu H., Murphy T.L., Ouyang W., Murphy K.M. (2001). Il-18-Stimulated Gadd45 Beta Required in Cytokine-Induced, but Not Tcr-Induced, Ifn-Gamma Production. Nat. Immunol..

[14] Zazzeroni F., Papa S., Algeciras-Schimnich A., Alvarez K., Melis T., Bubici C., Majewski N., Hay N., de Smaele E., Peter M.E. (2003). Gadd45 Beta Mediates the Protective Effects of Cd40 Costimulation against Fas-Induced Apoptosis. Blood.

[15] McInnes I.B., Schett G. (2007). Cytokines in the Pathogenesis of Rheumatoid Arthritis. Nat. Rev. Immunol..

[16] Bingham C.O. (2002). The Pathogenesis of Rheumatoid Arthritis: Pivotal Cytokines Involved in Bone Degradation and Inflammation. J. Rheumatol. Suppl..

[17] Brennan F.M., Hayes A.L., Ciesielski C.J., Green P., Foxwell B.M., Feldmann M. (2002). Evidence That Rheumatoid Arthritis Synovial T Cells Are Similar to Cytokine-Activated T Cells: Involvement of Phosphatidylinositol 3-Kinase and Nuclear Factor Kappab Pathways in Tumor Necrosis Factor Alpha Production in Rheumatoid Arthritis. Arthritis Rheumatol..

[18] Firestein G.S. (2003). Evolving Concepts of Rheumatoid Arthritis. Nature.

[19] Aletaha D., Neogi T., Silman A.J., Funovits J., Felson D.T., Bingham C.O., Birnbaum N.S., Burmester G.R., Bykerk V.P., Cohen M.D. (2010). 2010 Rheumatoid Arthritis Classification Criteria: An American College of Rheumatology/European League against Rheumatism Collaborative Initiative. Arthritis Rheumatol..

[20] Sultan F.A., Wang J., Tront J., Liebermann D.A., Sweatt J.D. (2012). Genetic Deletion of Gadd45b, a Regulator of Active DNA Demethylation, Enhances Long-Term Memory and Synaptic Plasticity. J. Neurosci. Off. J. Soc. Neurosci..

[21] Radonic A., Thulke S., Mackay I.M., Landt O., Siegert W., Nitsche A. (2004). Guideline to Reference Gene Selection for Quantitative Real-Time PCR. Biochem. Biophys. Res. Commun..

[22] Harrison P., Pointon J.J., Chapman K., Roddam A., Wordsworth B.P. (2008). Interleukin-1 Promoter Region Polymorphism Role in Rheumatoid Arthritis: A Meta-Analysis of Il-1b-511a/G Variant Reveals Association with Rheumatoid Arthritis. Rheumatology.

[23] Li R.N., Hung Y.H., Lin C.H., Chen Y.H., Yen J.H. (2010). Inhibitor Ikappabalpha Promoter Functional Polymorphisms in Patients with Rheumatoid Arthritis. J. Clin. Immunol..

[24] Lin C.H., Wang S.C., Ou T.T., Li R.N., Tsai W.C., Liu H.W., Yen J.H. (2008). I Kappa B Alpha Promoter Polymorphisms in Patients with Systemic Lupus Erythematosus. J. Clin. Immunol..

[25] Sweeney S.E., Firestein G.S. (2004). Signal Transduction in Rheumatoid Arthritis. Curr. Opin. Rheumatol..

[26] Heinrich P.C., Behrmann I., Haan S., Hermanns H.M., Muller-Newen G., Schaper F. (2003). Principles of Interleukin (Il)-6-Type Cytokine Signalling and Its Regulation. Biochem. J..

[27] Hintzen C., Quaiser S., Pap T., Heinrich P.C., Hermanns H.M. (2009). Induction of Ccl13 Expression in Synovial Fibroblasts Highlights a Significant Role of Oncostatin M in Rheumatoid Arthritis. Arthritis Rheumatol..

[28] Migita K., Komori A., Torigoshi T., Maeda Y., Izumi Y., Jiuchi Y., Miyashita T., Nakamura M., Motokawa S., Ishibashi H. (2011). Cp690,550 Inhibits Oncostatin M-Induced Jak/Stat Signaling Pathway in Rheumatoid Synoviocytes. Arthritis Res. Ther..

[29] Du F., Wang L., Zhang Y., Jiang W., Sheng H., Cao Q., Wu J., Shen B., Shen T., Zhang J.Z. (2008). Role of Gadd45 Beta in the Regulation of Synovial Fluid T Cell Apoptosis in Rheumatoid Arthritis. Clin. Immunol..

[30] Luo Y., Boyle D.L., Hammaker D., Edgar M., Franzoso G., Firestein G.S. (2011). Suppression of Collagen-Induced Arthritis in Growth Arrest and DNA Damage-Inducible Protein 45beta-Deficient Mice. Arthritis Rheumatol..

[31] De Groof A., Ducreux J., Humby F., Toukap A.N., Badot V., Pitzalis C., Houssiau F.A., Durez P., Lauwerys B.R. (2016). Higher Expression of Tnfalpha-Induced Genes in the Synovium of Patients with Early Rheumatoid Arthritis Correlates with Disease Activity, and Predicts Absence of Response to First Line Therapy. Arthritis Res. Ther..

[32] Zhang R.P., Shao J.Z., Xiang L.X. (2011). Gadd45a Protein Plays an Essential Role in Active DNA Demethylation During Terminal Osteogenic Differentiation of Adipose-Derived Mesenchymal Stem Cells. J. Biol. Chem..

